# Absence of Gamma-Interferon-Inducible Lysosomal Thiol Reductase (GILT) Is Associated with Poor Disease-Free Survival in Breast Cancer Patients

**DOI:** 10.1371/journal.pone.0109449

**Published:** 2014-10-21

**Authors:** Yu-Juan Xiang, Ming-Ming Guo, Cheng-Jun Zhou, Lu Liu, Bo Han, Ling-Yu Kong, Zhong-Cheng Gao, Zhong-Bing Ma, Lu Wang, Man Feng, Hai-Ying Chen, Guo-Tao Jia, De-Zong Gao, Qiang Zhang, Liang Li, Yu-Yang Li, Zhi-Gang Yu

**Affiliations:** 1 Department of Breast Surgery, The Second Hospital of Shandong University, Jinan, Shandong province, People's Republic of China; 2 Department of Pathology, The Second Hospital of Shandong University, Jinan, Shandong province, People's Republic of China; 3 Department of Pathology, Shandong University Medical School, Jinan, Shandong province, People's Republic of China; 4 Department of Breast Diseases, Linyi Tumor Hospital, Linyi, Shandong province, People's Republic of China; 5 Department of General Surgery, Linyi People's Hospital, Linyi, Shandong province, People's Republic of China; 6 Division of Epidemiology and Biostatistics, School of Public Health, Shandong University, Jinan, Shandong province, People's Republic of China; 7 Department of Pathology, Affiliated Hospital of Shandong Academy of Medical Sciences, Jinan, Shandong province, People's Republic of China; 8 Oral Maxillofacial-Head and Neck Key Laboratory of Medical Biology, and Central Laboratory for Experimental Medicine, Liaocheng People's Hospital, Liaocheng, Shandong province, People's Republic of China; 9 Department of Pathology, Liaocheng People's Hospital, Liaocheng, Shandong province, People's Republic of China; University of Alabama at Birmingham, United States of America

## Abstract

Tumor immunosurveillance is known to be of critical importance in controlling tumorigenesis and progression in various cancers. The role of gamma-interferon-inducible lysosomal thiol reductase (GILT) in tumor immunosurveillance has recently been studied in several malignant diseases, but its role in breast cancer remains to be elucidated. In the present study, we found GILT as a significant different expressed gene by cDNA microarray analysis. To further determine the role of GILT in breast cancer, we examined GILT expression in breast cancers as well as noncancerous breast tissues by immunohistochemistry and real-time PCR, and assessed its association with clinicopathologic characteristics and patient outcome. The absence of GILT expression increased significantly from 2.02% (2/99) in noncancerous breast tissues to 15.6% (34/218) in breast cancer tissues (*P*<0.001). In accordance with its proliferation inhibiting function, GILT expression was inversely correlated with Ki67 index (*P*<0.05). In addition, absence of GILT was positively correlated with adverse characteristics of breast cancers, such as histological type, tumor size, lymph nodes status, and pTNM stage (*P*<0.05). Consistently, breast cancers with reduced GILT expression had poorer disease-free survival (*P*<0.005). Moreover, significantly decreased expression of GILT was found in both primary and metastatic breast cancer cells, in contrast to normal epithelial cells. These findings indicate that GILT may act as a tumor suppressor in breast cancer, in line with its previously suggested role in anti-tumor immunity. Thus, GILT has the potential to be a novel independent prognostic factor in breast cancer and further studies are needed to illustrate the underlying mechanism of this relationship.

## Introduction

Breast cancer is one of the most common malignant diseases worldwide and the leading cause of cancer-related death in women [1]. Recurrence and metastasis are the main causes of death from this disease, and axillary lymph nodes invasion is the most important prognostic factor [2]. However, 22% to 33% of the patients without any detectable lymph node involvement, thought to be at low risk, develop recurrent disease after a 10-year follow-up [3]. Moreover, our previous study has indicated that the age of onset for breast cancer in Chinese women is declining [4]. Therefore, there is a great need to identify novel and reliable prognostic factors and therapeutic targets for breast cancer, which could be used to identify patients at high risk for poor prognosis and classify patients into suitable therapy strategies.

IFI30, also known as gamma-interferon-inducible lysosomal thiol reductase (GILT), the only thiol reductase localized in lysosomes and phagosomes [5], is a unique member of the thiol reductase family because its optimal pH is 4.5–5.5 [6], [7], [8]. GILT is synthesized as a precursor and processed into the matured form, a soluble glycoprotein, in the endosomal/lysosomal system [9]. GILT is constitutively expressed in professional antigen presenting cells (APCs), but in other cell types it is induced by inflammatory cytokines, such as interferon (IFN)-γ, interleukin (IL)-1β, and tumor necrosis factor (TNF)-α [9], [10]. GILT plays a pivotal role in exogenous antigen processing and presentation by catalyzing the reduction of the disulfide bonds of proteins [11], and expression levels can affect immune response to tumor antigens [12]. Previous studies have confirmed that interferon regulated gene-associated host responses (including GILT) play a central role in tumor immunosurveillance in skin [13]. Furthermore, it plays an essential role in regulating CD4+ T-cell tolerance to endogenous skin-restricted antigens related to generating effective immunotherapy for melanoma [14]. Although accumulated evidence suggests that GILT plays important roles in tumor immunosurveillance, its role in breast cancer is poorly understood.

In the present study, we found that GILT was significantly up-regulated in breast cancer tissues compared with adjacent, uninvolved tissues as revealed by cDNA microarray analyses. To further investigate the role of GILT in breast cancer pathogenesis, we used immunohistochemistry to evaluate the expression of GILT in relation to clinicopathologic characteristics and patient outcome. Our results demonstrated that loss of GILT expression was significantly associated with a worse disease-free survival in patients with breast cancer. Furthermore, we identified its potential role in tumor progression, as GILT expression decreased in primary cancer cells and metastatic cells compared with normal epithelial cells. We also found that GILT was an independent breast cancer prognostic factor.

## Materials and Methods

### Ethics Statement

This study was approved by the Institutional Review Board of the Second Hospital of Shandong University, Linyi People's Hospital and Linyi Tumor Hospital. Written informed consent was also obtained from each patient.

### Patients

All eligible specimens were collected from patients with pathologically and clinically confirmed breast cancer who underwent surgical resection prior to any therapy from January 1, 2005 to June 31, 2012. All samples were reevaluated by pathologists to confirm the diagnosis and estimate the tumor cell content. Adjacent, uninvolved breast tissue was obtained from breast cancer patients who underwent modified radical mastectomy; uninvolved parts referred to the regions more than 5 cm from the tumor sites. All frozen samples were obtained from the Department of Breast Surgery of the Second Hospital of Shandong University, and formalin-fixed paraffin-embedded tissue blocks were obtained from the Department of Pathology of the Second Hospital of Shandong University, Linyi People's Hospital and Linyi Tumor Hospital. Clinical follow-up was available for 218 cases.

### Gene expression microarray analyses

Total RNA from frozen tissues were extracted with Trizol Reagent (Invitrogen, Gaithersburg, MD, USA) following the manufacturer's instructions. RNA concentration and purity were determined by ultraviolet spectrophotometer (Nanodrop, ND-1000) by A260 and A260/280 ratio, and checked by electrophoresis on a 1.5% agarose/formaldehyde gel. The human long oligonucleotide microarray was constructed by CapitalBio Corporation (Beijing, People's Republic of China). The microarray consists of 5′-amino-modified 70-mer probes representing 35035 well-characterized human genes purchased from Operon Company.

### Immunohistochemical analyses

Streptavidin-peroxidase-biotin (SP) immunohistochemical method was performed to study the expression of GILT. After deparaffinization and rehydration, tissue sections were incubated in 3% hydrogen peroxide in methanol to quench the endogenous peroxidase activity, followed by incubation with normal serum to block nonspecific binding. The sections were incubated with Rabbit anti-GILT (1∶400; HPA026650, Sigma-Aldrich Corp, St Louis, MI, USA) overnight at 4°C, and then incubated with a secondary antibody from the SP reagent kit (Zhongshan Biotechnology Company, PV9000, Beijing, People's Republic of China). Slides were stained with diaminobenzidine (DAB; Zhongshan Biotechnology Company), counterstained with hematoxylin, dehydrated, treated with xylene, and mounted. For negative controls, the rabbit anti-GILT antibody was replaced with phosphate buffer solution.

### Evaluation of immunohistochemical staining

The stained slides were reviewed and scored independently by two pathologists blinded to patient information, and the scores were determined by combining the proportion of positively stained tumor cells and the intensity of staining [15]. Briefly, five views were examined per slide, and 100 cells were observed per view at 400× magnification. Initially, a proportion score was assigned, which represented the estimated proportion of positive tumor cells (0, none; 1, <1/100; 2, 1/100 to 1/10; 3, 1/10 to 1/3; 4, 1/3 to 2/3; and 5,>2/3). If the proportion scores were ≤3, we considered these “low” staining, otherwise cases were considered “high” staining. Next, an intensity score was assigned, which represented the average staining intensity of the positive tumor cells (0, none; 1, weak; 2, intermediate; and 3, strong). If the intensity score was 0, we classified it as GILT negative;>0 and we classified it as GILT positive. The proportion and intensity scores were then added to obtain a total score, which ranged from 0 to 8. Total scores ≤4 were considered as low expression; >4 was considered high expression.

HER2 immunostaining was evaluated as 0, 1+, 2+, and 3+ according to ASCO guidelines [16]. Tumors with 0 and 1+ staining were considered negative, and tumors with 3+ staining were considered positive. If the staining was evaluated as 2+, fluorescence in situ hybridization was used to determine the final status. For Ki67 index, high expression was defined as >14% of tumor cells showing moderate to strong immunoreactivity [17].

### Laser-capture microdissection (LCM)

Frozen samples were mounted in opti-mum cutting temperature (OCT) compound and frozen, serial 8 µm sections were cut using a cryostat microtome (Leica CM, Leica Microsystems, Wetzlar, Germany) at −20°C, and placed onto prepared membrane slides (PEN membrane covered, Leica Microsystems). Tissues were rehydrated with decreasing concentrations of ethanol and then stained for 40 seconds with 300 µL of Cresyl violet (LCM Staining Kit, Life Technologies, Carlsbad, CA, USA), and then dehydrated and treated with xylene. Under microscopic observation, parts of cancer cell nests as well as normal epithelial cells were microdissected using the AS-LCM System (Leica Microsystems). The harvested cells were stored in 700 µL QIAzol Lysis Reagent (miRNeasy Micro kit, Qiagen, Limburg, Netherlands) at −80°C for further RNA extraction.

### RNA extraction and real-time reverse transcriptase-PCR assay

Total RNA was extracted according to the miRNeasy Micro Kit (Qiagen) manufacturer's protocol from cells harvested by LCM. Total RNA was dissolved in 14 µL of diethyl pyrocarbonate-treated H_2_O. All RNA was verified for purity by measuring the ratios of the absorbance at 260 nm and 280 nm (A260/A280) using a spectrophotometer. All the RNA was reverse-transcribed in a final volume of 20 µL using a PrimeScript RT reagent Kit with gDNA Eraser (TaKaRa Bio Inc, Shiga, Japan) according to the protocol. Quantitative analysis of GILT mRNA expression was performed in paired breast cancer cells and normal epithelial cells using the RealSYBR Mixture (CWBIO, Beijing, People's Republic of China). GILT was amplified using the following primers: 5′-TGACCCTCTACTATGAAGCACTG-3′ (forward primer) and 5′- CCACTGACATTTTGTTCCTGTG-3′ (reverse primer). ACTB was used as an endogenous control with the following primers: 5′-TGACGTGGACATCCGCAAAG -3′ (forward primer) and 5′-CTGGAAGGTGGACAGCGAGG-3′ (reverse primer). The results were evaluated by the comparative threshold cycle value method (2^−Δct^) for relative quantification. Each reverse transcriptase (RT)-qPCR experiment was repeated in triplicate.

### Statistical analysis

Differences in GILT expression (intensity, proportion, and total score) between breast cancer and noncancerous breast tissue were evaluated by Chi-square test. Differences between negative and positive GILT expression group regarding clinicopathologic characteristics were evaluated by Chi-square test or Fisher's exact test, when a Chi-square test was conducted with cells that had an expected frequency of five or less. Using the same methods, the correlation between different proportion scores, different total scores, and clinicopathologic characteristics, if appropriate, were also studied. Kaplan–Meier method and log-rank test were conducted to evaluate the influence of GILT expression on disease-free survival (DFS) of patients. Furthermore, hazard ratios (HRs) and 95% confidential intervals (CIs) were computed from multivariate Cox regression models. Data from real-time PCR were expressed as means ± standard deviation (SD). Statistical significance was evaluated with the Student's *t-*test. *P*<0.05 was considered significant, and all tests were two-sided. All statistical analyses were performed using the statistical software package SAS 9.1.3 (SAS Institute, Cary, NC, USA).

## Results

### Differential gene expression profiles between breast cancer tissues and normal breast tissues

To look for new, promising oncogenic genes, gene expression profiles of seven pairs of matched frozen breast cancer and noncancerous tissue samples were analyzed using cDNA microarrays. Among the 427 significantly different expressed genes (*P*<0.05), we identified 221 up-regulated genes and 206 down-regulated genes in breast cancer tissues compared with their normal counterparts. These genes could be divided into several classes based on their functions, some of which were related to immunity (e.g., GILT, HLA-B, HLA-H, TAP1, CX3CL1, PIK3R1, and CXCL12). The up-regulated genes that exhibit more than five-fold changes between cancer and normal tissues are shown in Table 1. The role of GILT in tumor immunology provides rationale for exploring its function in breast cancer; to date, there are no detailed studies regarding this gene and breast cancer. Thus, we selected GILT (fold change  = 5.4016, *P*<0.001) as a potential biomarker to investigate in breast cancer.

**Table 1:** Selected significantly up-regulated genes when comparing breast cancer tissue with normal breast tissue by cDNA microarray.

Accession	Gene symbol	Description	Fold change	*P* value
NM_006332.4	IFI30	Gamma-interferon-inducible lysosomal thiol reductase precursor	5.4016	<0.001
NM_005192.2	CDKN3	Cyclin-dependent kinase inhibitor 3	5.1270	<0.001
NR_002734.1	PTTG3	pituitary tumor-transforming 3	5.5751	<0.001
NM_012112.4	TPX2	Targeting protein for Xklp2	9.6235	<0.001
NM_001034.1	RRM2	Ribonucleoside-diphosphate reductase M2 subunit	9.1722	<0.001
NM_001827.1	CKS2	Cyclin-dependent kinases regulatory subunit 2	5.0202	<0.001
NM_005101.1	ISG15	Interferon-induced 17 kDa protein precursor	7.4359	<0.001
NM_002351.2	SH2D1A	SH2 domain-containing protein 1A	6.3158	<0.001
NM_014176.1	UBE2T	Ubiquitin-conjugating enzyme E2 T	6.0612	<0.001
NM_004219.3	PTTG1	Securin	5.0617	<0.001
NM_005980.2	S100P	Protein S100-P	9.7325	<0.001
NM_014736.4	KIAA0101	PCNA-associated factor	11.1746	0.0069
NM_020675.3	SPBC25	Kinetochore protein Spc25	5.3107	0.0069
NM_000088.3	COL1A1	Collagen alpha-1(I) chain precursor.	8.4532	0.0069
NM_001168.2	BIRC5	Baculoviral IAP repeat-containing protein 5	6.7460	0.0069
NM_004217.2	AURKB	Serine/threonine-protein kinase 12	5.3192	0.0110
NM_031423.3	NUF2	Kinetochore protein Nuf2	9.9257	0.0110
NM_001237.2	CCNA2	Cyclin-A2	7.8825	0.0125
NM_004701.2	CCNB2	G2/mitotic-specific cyclin-B2	8.7084	0.0125
NM_033379.2	CDC2	Cell division control protein 2 homolog	13.5476	0.0125
NM_004336.2	BUB1	Mitotic checkpoint serine/threonine-protein kinase BUB1	7.5387	0.0125
NM_002466.2	MYBL2	Myb-related protein B	7.5657	0.0142
NM_016343.3	CENPF	centromere protein F	5.0906	0.0166
NM_002133.1	HMOX1	Heme oxygenase 1	7.5513	0.0210
NM_003129.3	SQLE	Squalene monooxygenase	5.2500	0.0210
NM_003225.2	TFF1	Trefoil factor 1 precursor	6.4284	0.0210
NM_014750.3	DLG7	Discs large homolog 7	9.9318	0.0328
NM_016640.3	MRPS30	Mitochondrial 28S ribosomal protein S30	6.1910	0.0414
NM_001002800.1	SMC4	Structural maintenance of chromosomes protein 4	5.7334	0.0498

### Differential expression status of GILT in breast cancer tissues and noncancerous breast tissues

**Figure 1:**
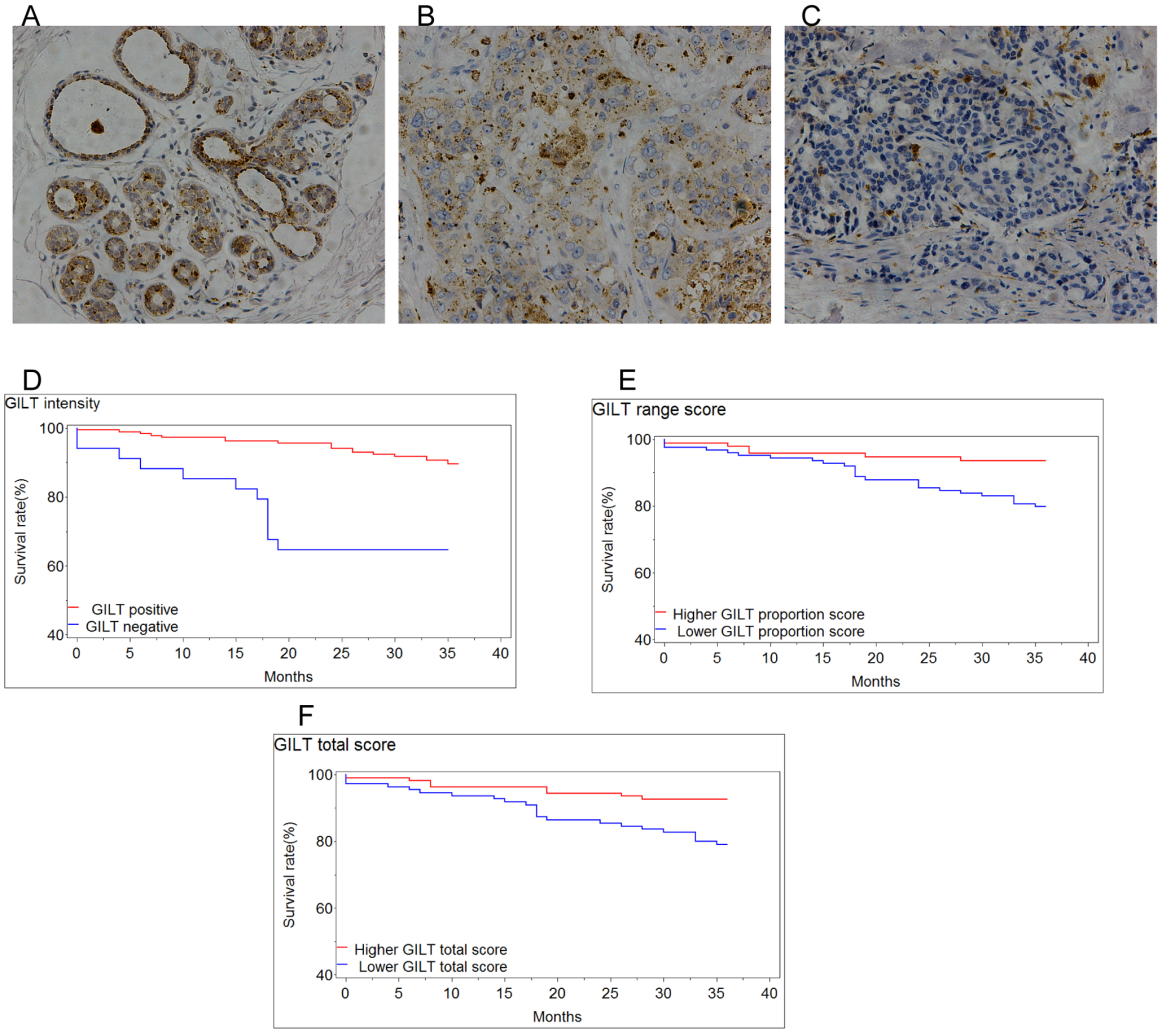
Expression of GILT and survival analysis in breast cancer. (A–C) GILT expression in breast tissues (original magnification, ×200). (A) Representative positive staining of GILT in normal breast tissue. (B) Representative positive staining of GILT in breast cancer tissues. (C) Representative negative staining of GILT in breast cancer tissues. (D–F) The expression status of GILT in breast cancer tissues were correlated with DFS by Kaplan–Meier estimates. (D) Decreased DFS time was observed in GILT negative patients (*P*<0.0001). (E) Decreased DFS time was observed in the lower GILT expression range score group (*P* = 0.0041). (F) Decreased DFS time was observed in the lower total GILT expression score group (*P* = 0.0041).

To determine whether the expression level of GILT differs between breast cancer tissues and noncancerous breast tissues, immunohistochemistry analysis of 218 breast cancer tissue sections and 99 noncancerous breast tissue sections was conducted. The GILT protein appeared to be expressed in the cytoplasmic component of breast cancer cells (Figure 1A, B, C). The negative expression rate of GILT increased significantly from 2.02% (2/99) in noncancerous breast tissues to 15.6% (34/218) in breast cancer tissues (*P*<0.001). This result suggests a potential role of the loss of GILT in breast cancer pathogenesis.

### Correlation between GILT expression and clinicopathologic characteristics

To assess the effect of GILT in breast cancer, we analyzed the association between GILT expression and prognosis-related pathological characteristics of 218 breast cancer patients. The expression status of GILT in cancer tissues correlated with certain key clinicopathologic characteristics (Table 2). For instance, the intensity of GILT expression in breast cancer tissues was significantly inversely correlated with Ki67 index (*P* = 0.0115) and recurrences at 3 years (*P* = 0.0001). The proportion of GILT expression was significantly correlated with histological type (*P* = 0.0421), tumor size (*P* = 0.0002), lymph node status (*P* = 0.0041), pTNM stage (*P* = 0.0016), Ki67 index (*P* = 0.0159), and recurrences at 3 years (*P* = 0.0039). In addition, the total GILT expression score was significantly correlated with histological type (*P* = 0.0270), tumor size (*P* = 0.0015), lymph node status (*P* = 0.0030), pTNM stage (*P* = 0.0061), Ki67 index (*P* = 0.0152), and recurrences at 3 years (*P* = 0.0043). These results indicate that decreased GILT expression was associated with clinicopathologic risk factors in breast cancer.

**Table 2:** Correlation between GILT staining (including intensity, proportion score, and total score) and clinicopathologic characteristics as well as DFS of breast cancer patients (n = 218).

	Intensity			Proportion score			Total score		
Characteristics	Positive (%)	Negative (%)	*P*	Higher(>3) (%)	Lower(≤3) (%)	*P*	Higher(>4) (%)	Lower(≤4) (%)	*P* a
**Age (year)**									
<45	68(36.96)	13(38.24)	0.82	32(34.04)	49(39.52)	0.59	37(34.26)	44(40.00)	0.58
45–60	90(48.91)	15(44.12)		46(48.94)	59(47.58)		53(49.07)	52(47.27)	
>60	26(14.13)	6(17.65)		16(17.02)	16(12.90)		18(16.67)	14(12.73)	
**Histological type**									
IDC	163(88.59)	31(91.18)	0.66	79(84.04)	115(92.74)	**0.04**	91(84.26)	103(93.64)	**0.03**
Other	21(11.41)	3(8.82)		15(15.96)	9(7.26)		17(15.74)	7(6.36)	
**Tumor size**									
T1	55(29.89)	7(20.59)	0.28	38(40.43)	24(19.35)	**<0.01**	39(36.11)	23(20.91)	**<0.01**
T2	100(54.35)	22(64.71)		39(41.49)	83(66.94)		49(45.37)	73(66.36)	
T3	17(9.24)	1(2.94)		12(12.77)	6(4.84)		14(12.96)	4(3.64)	
T4	12(6.52)	4(11.76)		5(5.32)	11(8.87)		6(5.56)	10(9.09)	
**Lymph nodes status**									
N0	101(54.89)	15(44.12)	0.52	62(65.96)	54(43.55)	**<0.01**	69(63.89)	47(42.73)	**<0.01**
N1	35(19.02)	9(26.47)		10(10.64)	34(27.42)		12(11.11)	32(29.09)	
N2	35(19.02)	6(17.65)		15(15.96)	26(20.97)		18(16.67)	23(20.91)	
N3	13(7.07)	4(11.76)		7(7.45)	10(8.06)		9(8.33)	8(7.27)	
**pTNM stage**									
0–I	44(23.91)	5(14.71)	0.46	32(34.04)	17(13.71)	**<0.01**	34(31.48)	15(13.64)	**0.01**
II	87(47.28)	17(50.00)		37(39.36)	67(54.03)		44(40.74)	60(54.55)	
III–IV	53(28.80)	12(35.29)		25(26.60)	40(32.26)		30(27.78)	35(31.82)	
**ER/PR**									
Positive b	130(70.65)	19(55.88)	0.09	68(72.34)	81(65.32)	0.27	78(72.22)	71(64.55)	0.22
Double negative	54(29.35)	15(44.12)		26(27.66)	43(34.68)		30(27.78)	39(35.45)	
**HER2**									
Negative	167(90.76)	31(91.18)	0.94	82(87.23)	116(93.55)	0.11	96(88.89)	102(92.73)	0.33
Positive	17(9.24)	3(8.82)		12(12.77)	8(6.45)		12(11.11)	8(7.27)	
**Ki67 index**									
0–14	123(66.85)	15(44.12)	**0.01**	68(72.34)	70(56.45)	**0.02**	77(71.30)	61(55.45)	**0.02**
≥14	61(33.15)	19(55.88)		26(27.66)	54(43.55)		31(28.70)	49(44.55)	
**DFS of 3 years** c									
No	165(89.67)	22(64.71)	**<0.01**	88(93.62)	99(79.84)	**<0.01**	100(92.59)	87(79.09)	**<0.01**
Yes	19(10.33)	12(35.29)		6(6.38)	25(20.16)		8(7.41)	23(20.91)	

Abbreviations: IDC, invasive ductal carcinoma; pTNM stage, pathological TNM stage; ER, estrogen receptor; PR, progesterone receptor; HER2, human epidermal growth factor receptor; DFS, disease-free survival.

a Chi-square test.

b Positive expression of hormone receptor, at least one of ER or PR was positive.

c Tumor recurrences and tumor-related deaths for positive events of DFS.

### Loss of GILT expression in breast cancer was associated with disease-free survival

GILT expression patterns in breast cancer tissues were further correlated with the disease-free survival by Kaplan–Meier estimates. Adverse disease-free survival was observed in GILT expression negative patients (*P*<0.0001, Figure 1D), lower GILT proportion score group (*P* = 0.0041, Figure 1E) and the lower total GILT staining score group (*P* = 0.0041, Figure 1F), as determined by the log-rank test.

To further evaluate whether loss of GILT was an independent prognostic factor for patients with breast cancer, multivariate Cox proportional hazard regression analyses were conducted. As shown in Tables 3 and 4, in 218 primary breast cancer patients, our results showed that similarly to pTNM stage, which had been widely used as an important prognostic factor for survival of patients with breast cancer, negative GILT expression (HR  = 3.372, 95% CI 1.212–9.384, *P* = 0.0199) as well as lower total GILT staining score (HR  = 2.963, 95% CI1.109–1.917, *P* = 0.0303) was a significant independent adverse predictor for disease-free survival.

**Table 3:** Multivariate Cox regression analysis on 3-year disease-free survival of 218 patients with breast cancer (including intensity and proportion of GILT expression).

Variable	HR	95%CI		?^2^	*P*
		Lower	Upper		
**Intensity of GILT**	**3.372**	**1.212**	**9.384**	**5.421**	**0.020**
Proportion of GILT	1.804	0.539	6.043	0.916	0.339
TIL score	2.474	0.938	6.524	3.353	0.067
Disease side	0.841	0.321	2.205	0.124	0.725
Age	1.089	0.423	2.803	0.032	0.859
**BMI** a	**2.569**	**1.370**	**4.816**	**8.656**	**0.003**
Menarche	3.389	0.660	17.400	2.139	0.144
Menopause	1.088	0.308	3.838	0.017	0.896
Hypertension	0.300	0.034	2.645	1.175	0.278
Diabetes mellitus	0.687	0.076	6.246	0.111	0.739
Peripheral blood neutrophils	2.187	0.571	8.383	1.303	0.254
Peripheral blood lymphocytes	1.356	0.451	4.079	0.294	0.588
Peripheral blood hemoglobin	1.423	0.454	4.467	0.366	0.545
Histological type	1.269	0.264	6.113	0.088	0.766
**pTNM stage**	**4.343**	**1.913**	**9.860**	**12.319**	**<0.001**
Molecular classification	1.372	0.971	1.939	3.208	0.073

Abbreviations: HR, hazard ratio; CI, confidence interval; TIL, tumor infiltrating lymphocyte; BMI, body mass index; pTNM stage, pathological TNM stage.

a the international BMI classification.

**Table 4:** Multivariate Cox regression analysis on 3-year disease-free survival of 218 patients with breast cancer (including total GILT expression score).

Variable	HR	95%CI		?^2^	*P*
		Lower	Upper		
**Total score of GILT**	**2.963**	**1.109**	**7.917**	**4.692**	**0.030**
TIL score	2.324	0.881	6.126	2.906	0.088
Disease side	0.805	0.318	2.037	0.211	0.646
Age	1.249	0.465	3.354	0.195	0.659
**BMI** a	**2.517**	**1.301**	**4.872**	**7.508**	**0.006**
Menarche	2.505	0.512	12.257	1.285	0.257
Menopause	0.977	0.260	3.679	0.001	0.973
Hypertension	0.420	0.050	3.566	0.631	0.427
Diabetes mellitus	0.827	0.090	7.585	0.028	0.867
Peripheral blood neutrophils	2.000	0.510	7.842	0.988	0.320
Peripheral blood lymphocytes	1.068	0.367	3.110	0.015	0.903
Peripheral blood hemoglobin	1.242	0.408	3.775	0.146	0.703
Histological type	1.498	0.320	7.004	0.264	0.608
**pTNM stage**	**3.845**	**1.819**	**8.129**	**12.428**	**<0.001**
**Molecular classification**	**1.478**	**1.048**	**2.084**	**4.955**	**0.026**

Abbreviations: HR, hazard ratio; CI, confidence interval; TIL, tumor infiltrating lymphocyte; BMI, body mass index; pTNM stage, pathological TNM stage.

a the international BMI classification

**Figure 2:**
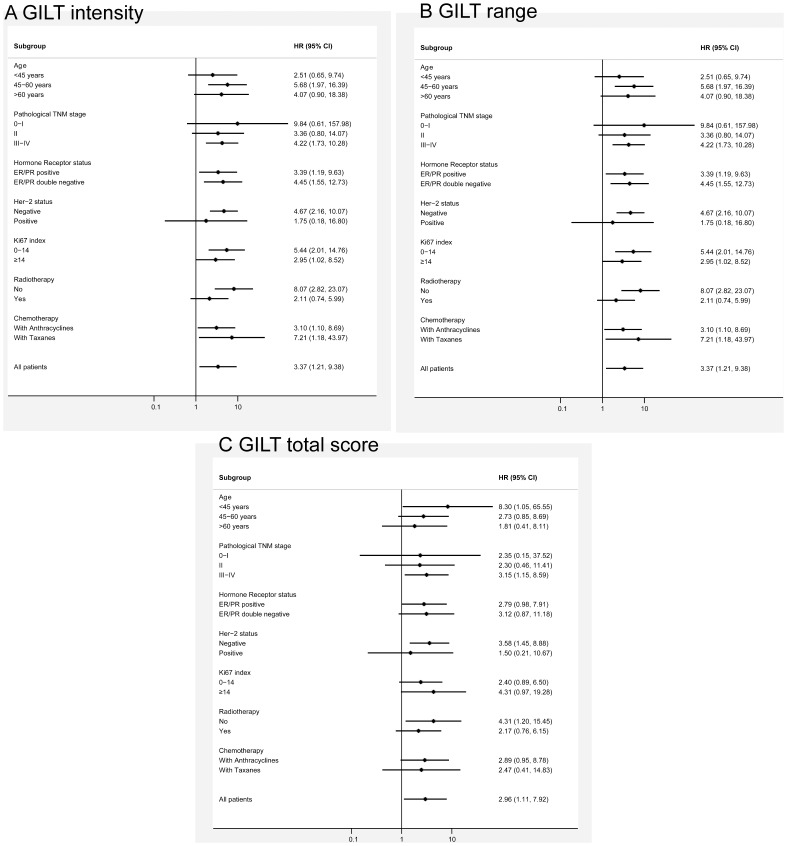
Subgroup analyses for DFS according to GILT expression. (A) Forest plots showing hazard ratios (and 95% confidence intervals) for disease-free survival for the intensity of GILT staining in subgroup analyses by clinicopathologic characteristics of patients. (B) Forest plots showing hazard ratios (and 95% confidence intervals) for disease-free survival for the proportion score of GILT staining in subgroup analyses by clinicopathologic characteristics of patients. (C) Forest plots showing hazard ratios (and 95% confidence intervals) for disease-free survival for the total score of GILT staining in subgroup analyses by clinicopathologic characteristics of patients. Disease-free survival favored higher GILT expression over decreased expression, including the intensity, proportion, as well as total score of GILT staining in all subgroups (HRs>1).

To determine a potential relationship between the prognostic value of GILT expression detected by immunohistochemistry and known clinicopathologic prognostic factors as well as treatment strategies, subgroup analyses for disease-free survival were conducted. As shown in Figure 2, disease-free survival favored higher GILT expression over decreased expression, including intensity, range, and total GILT staining score, in all subgroups (HRs>1).

### GILT mRNA level increased but GILT protein level decreased in breast cancer cells compared with normal epithelial cells

**Figure 3:**
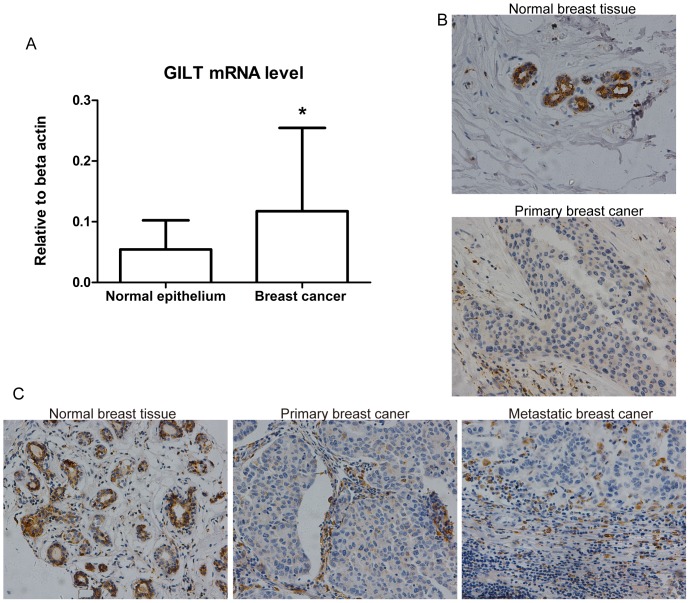
Involvement of GILT in tumorigenesis and lymph node metastasis in breast cancer. (A) GILT mRNA expression increased in malignant cells compared with adjacent normal epithelial cells. The real-time PCR results confirmed that there was a 2.18-fold up-regulation of GILT mRNA in breast cancer cells compared with adjacent normal epithelial cells (* *P*<0.05, n = 19). Relative means ± standard deviation for GILT mRNA obtained from tumor tissue and normal adjacent tissue are shown. (B) GILT protein expression decreased in malignant tissues compared with adjacent normal epithelial tissues. The immunohistochemistry results confirmed that 78.95% (15/19) showed weaker staining in carcinoma tissue than in adjacent normal breast tissue; representative images are shown (original magnification, ×200). (C) GILT protein expression in matched normal-cancerous-metastatic breast tissues (n = 44). Representative GILT expression in respective normal-cancerous-metastatic breast tissues sections from one patient. Both intensity and proportion score of GILT staining in primary breast cancer as well as metastatic cancer tissue were 0, compared with 2 and 4 respectively in normal breast tissue (original magnification, ×200).

To validate changes in GILT mRNA and protein when transforming from normal epithelial cells to cancerous cells, real-time PCR as well as immunohistochemistry was carried out in 19 breast cancer patients. To eliminate the influence of the constitutive expression of GILT in fibroblasts, which were abundant in breast stroma, LCM was employed to obtain a relatively pure population of cells for mRNA evaluation. As shown in Figure 3A, real-time PCR results confirmed that GILT mRNA increased (2.18-fold change) in breast cancer cells compared with corresponding adjacent normal epithelial cells (*P* = 0.0427). However, the intensity (*P* = 0.0059), proportion (*P* = 0.0082), and total score (*P* = 0.0027) of GILT expression decreased significantly in cancerous tissue compared with normal tissue (Table 5; Figure 3B). Taken together, GILT significantly increased at the mRNA level, but decreased at the protein level in breast cancer cells compared with normal epithelial cells, indicating its potential role in breast cancer tumorigenesis.

**Table 5:** GILT expression was significantly down-regulated in breast cancer tissues compared with normal epithelial tissues (n = 19).

GILT expression	Normal breast tissue (%)	Breast cancer tissue (%)	S	*P*
Intensity				
0	0(0.00)	0(0.00)	−33	**0.0059** a
1	4(21.05)	14(73.69)		
2	13(68.42)	4(21.05)		
3	2(10.53)	1(5.26)		
Proportion score				
≥3	14(73.68)	7(36.84)	7	**0.0082** b
0–2	5(26.32)	12(63.16)		
Total score				
≥4	16(84.21)	7(36.84)	9	**0.0027** c
0–3	3(15.79)	12(63.16)		

a Wilcoxon signed rank test.

b Chi-square test.

c Chi-square test.

### GILT expression decreases with breast cancer development from normal to primary and metastatic cancers

To investigate the effects of GILT on breast cancer pathogenesis, immunohistochemistry was conducted on the 44 matched normal-cancerous-metastatic tissue samples. There was a significant difference in the expression intensity of GILT among the three groups (*P* = 0.0017); both primary breast cancer tissues (*P = *0.0011) and metastatic tissues (*P = *0.0261) presented significant lower GILT expression than normal breast tissues, but there was no significant difference in GILT expression between primary tumor tissues and metastatic tissues. Representative immunohistochemical images are shown in Figure 3C. The result demonstrated the potential role of GILT in breast cancer pathogenesis. (All the original experiment results were presented in “Supporting Information S1”).

## Discussion

Tumor immunosurveillance is known to be of critical importance in controlling tumorigenesis and progression in various cancers [18], [19], [20]. Recently, the role of GILT in tumor immunosurveillance has been studied in several malignant diseases. In this study, we used cDNA microarray analyses, immunohistochemistry and real-time RT-PCR to evaluate GILT expression in noncancerous as well as breast cancer tissues, its effect on patient outcome, and its potential role in breast cancer pathogenesis. Our results demonstrate that GILT expression is significantly decreased in breast cancer pathogenesis. Furthermore, GILT expression correlates with certain central prognostic pathological characteristics of breast cancer, as well as disease-free survival, and additionally serves as an independent prognostic factor for breast cancer. To our knowledge, this study is the first to characterize GILT roles in breast cancer and thus represents a promising potential for establishing GILT as a prognostic predictor and therapeutic target.

In this study, our finding that GILT expression decreases in cancerous cells compared with normal controls is unprecedented in breast cancer. Similar to the observation of the present study, previous research has discovered that GILT was absent or expressed at greatly reduced levels in several malignant diseases, such as melanomas [12], [21], prostate cancer [22] and glioblastoma [23], indicating a potential role of GILT in tumorigenesis.

A reduction in or the absence of GILT expression in cancer tissues correlates with poor prognosis in patients with breast cancer, and represents a large possibility of recurrence as early as 3 years after diagnosis. Additionally, an inverse relationship between GILT expression and Ki67 index, a proliferation marker for breast cancer [24], is in agreement with the cell proliferation inhibiting role of GILT in fibroblasts [25], which functions by affecting activity and stability of superoxide dismutase (SOD2), and consequently affects the levels of reactive oxygen radicals (ROS) and ERK1/2 phosphorylation [25], [26], [27]. Several recent studies have demonstrated that higher Ki67 proliferative activity was associated with poorer prognosis with respect to recurrence-free survival [28], [29], [30], [31], cancer-specific survival [30], [32], [33], and clinical response to chemotherapy [28], [34]. The data herein demonstrates that reduced expression of GILT is associated with high Ki67 expression; thus, cancers with high proliferation, may consequently lead to early recurrence. Moreover, GILT expression inversely correlates with adverse clinicopathologic characteristics, including tumor size, lymph node status, as well as pathological TNM stage of breast cancer. Therefore, these observations support the adverse prognostic implication of GILT absence in breast cancer. It should be pointed out, however, that our current analysis evaluates disease-free survival but does not consider overall survival, which remains to be tested in the future.

In agreement with its known role as an important enzyme affecting immune responses to tumors [10], [12], [21], [35], the absence of GILT shows a potential pro-tumor role in breast cancer, because decreased GILT expression are observed both in primary and metastatic cancer cells compared with their adjacent normal epithelial cells. One of the possible mechanisms concerning the role of GILT in cancers is thought to be related to the processing and presentation of tumor antigens [12], [21], [23]. As the only known thiol reductase in the endocytic compartment [8], the absence of GILT fails to generate MHC class II associated epitopes that require disulfide bond reduction, which are proteins involved in apoptosis, mitosis regulation, and transcription factors [36], [37], as well as cross-presentation of antigen through the MHC class I restricted pathway [5]. Together, this results in a reduction in T cell stimulation, and recognition failure of some antigens by effector T cells. Malignant cells may take advantage of this mechanism to hide and escape from host immune system surveillance and clearance. Therefore, our results warrant further research to verify GILT's true function in tumor immune responses in breast cancer, and targeted GILT induction may be a promising approach for breast cancer treatment in the future.

Furthermore, we also found that GILT mRNA level was up-regulated in breast cancer cells compared with normal cells, in agreement with its role in gene expression predictor of breast cancer outcomes which exhibits higher GILT mRNA levels in metastatic than nonmetastatic forms [38]. However, GILT protein level was down-regulated in cancer cells compared with adjacent normal epithelial cells. This conflicting observation between mRNA and protein levels of GILT in breast cancer was quite different from what have found in Diffuse Large B-Cell Lymphoma (DLBCL), which demonstrated that variation in GILT protein expression correlated strongly with its mRNA expression within tumor cells [39]. Although studies on the GILT accumulated in recent years, the mechanism for regulating GILT expression was somewhat inconsistent in different cell types. For example, constitutively expressed GILT was negative regulated by STAT1 in fibroblasts [40], while in melanoma, it was induced directly by STAT1 in IFN-γ-inducible expression way [41]. And also, GILT may be dramatically induced by Egr-1 in endothelial cells [42]. Thus, the inconsistent findings in GILT mRNA and protein expression between breast cancer and DLBCL may be due to the different origin of these two cancer types. What's more, we have demonstrated preliminarily that there was post-transcriptional regulation of GILT expression in breast cancer cells. GILT protein may be degraded by proteasomes, because the GILT protein level increased significantly after treated with proteasome inhibitor MG-132 in MCF-7 cells (The results were shown in Figure S1, and the original western blotting photos were presented in “Supporting Information S2”). However, these results are far from being able to explain the current findings. The exact mechanism of the contradictory regulation of GILT between mRNA and protein levels in the pathogenesis of breast cancer needs to be further clarified in future studies.

In conclusion, the absence of GILT expression in primary breast cancer was independently associated with poor disease-free survival of patients. Therefore, it has the potential to be a novel characteristic to be taken into consideration to sub-classify breast cancer with respect to prognosis as well as to determine individualized treatment strategies. Moreover, its potential roles in breast cancer pathogenesis we identified in the present study provide the rationale and direction for continued studies on GILT to elucidate its true function in breast cancer.

## Supporting Information

Figure S1 GILT protein changed in MCF-7 cells after treated with proteasomes inhibitor MG-132. (A) Flow cytometry (FCM) analysis of the changes of GILT protein level in breast cancer cells after threated with 10 µM MG-132 for 12 hours in MCF-7 cells. (B) The positive expression rate of GILT detected by FCS in cancer cells significantly increased from 51.7% to 73.6% after treated with MG-132 for 12 hours (P = 0.044). (C) Western blot analysis of the changes of GILT protein expression in breast cancer cells after threated with 10 µM MG-132 for 12 hours in MCF-7 cells. (D) Protein band density was analyzed with the Image J software. β-actin was used as the internal control. Data was normalized and expressed as means ± standard deviation (SD). And the relative protein expression level of GILT in cancer cells significantly increased after treated with MG-132 for 12 hours (P = 0.008).
(TIF)

Supporting Information S1 All original experiment datasets.
(RAR)

Supporting Information S2 Original western blotting photos.
(RAR)
