# Correction: Absence of Gamma-Interferon-Inducible Lysosomal Thiol Reductase (GILT) Is Associated with Poor Disease-Free Survival in Breast Cancer Patients

**DOI:** 10.1371/journal.pone.0117653

**Published:** 2015-01-28

**Authors:** The PLOS ONE Staff

There is an error in Fig. 2. The authors have provided a corrected figure below.

**Figure 2:**
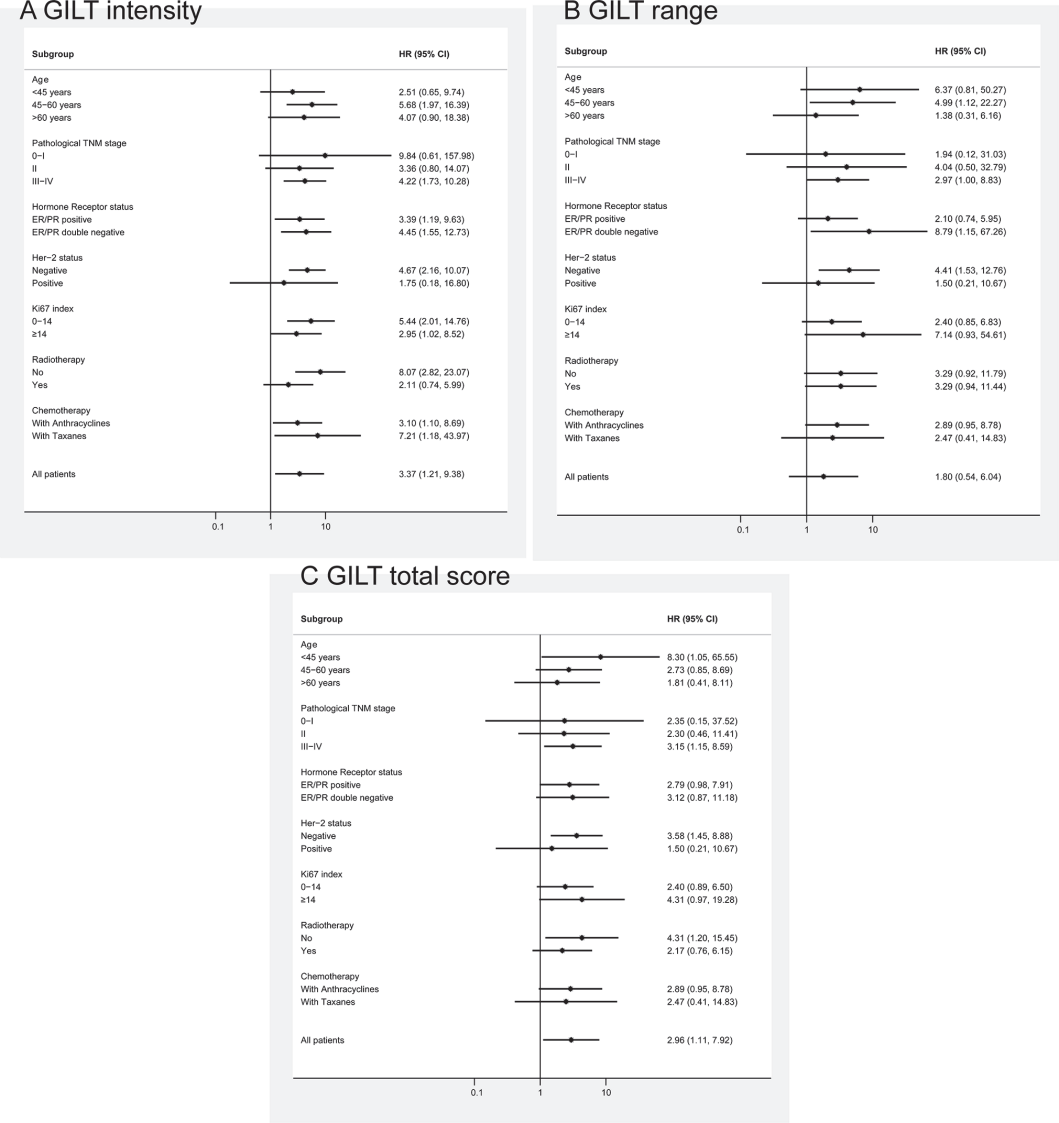
Subgroup analyses for DFS according to GILT expression. (A) Forest plots showing hazard ratios (and 95% confidence intervals) for disease-free survival for the intensity of GILT staining in subgroup analyses by clinicopathologic characteristics of patients. (B) Forest plots showing hazard ratios (and 95% confidence intervals) for disease-free survival for the proportion score of GILT staining in subgroup analyses by clinicopathologic characteristics of patients. (C) Forest plots showing hazard ratios (and 95% confidence intervals) for disease-free survival for the total score of GILT staining in subgroup analyses by clinicopathologic characteristics of patients. Disease-free survival favored higher GILT expression over decreased expression, including the intensity, proportion, as well as total score of GILT staining in all subgroups (HRs>1).
