# Supplementary figures and images for: Absence of Gamma-Interferon-Inducible Lysosomal Thiol Reductase (GILT) Is Associated with Poor Disease-Free Survival in Breast Cancer Patients
Source: PLoS One. 2014 Oct 21;9(10):e109449. doi: 10.1371/journal.pone.0109449 (PMC4204821; doi:10.1371/journal.pone.0109449)

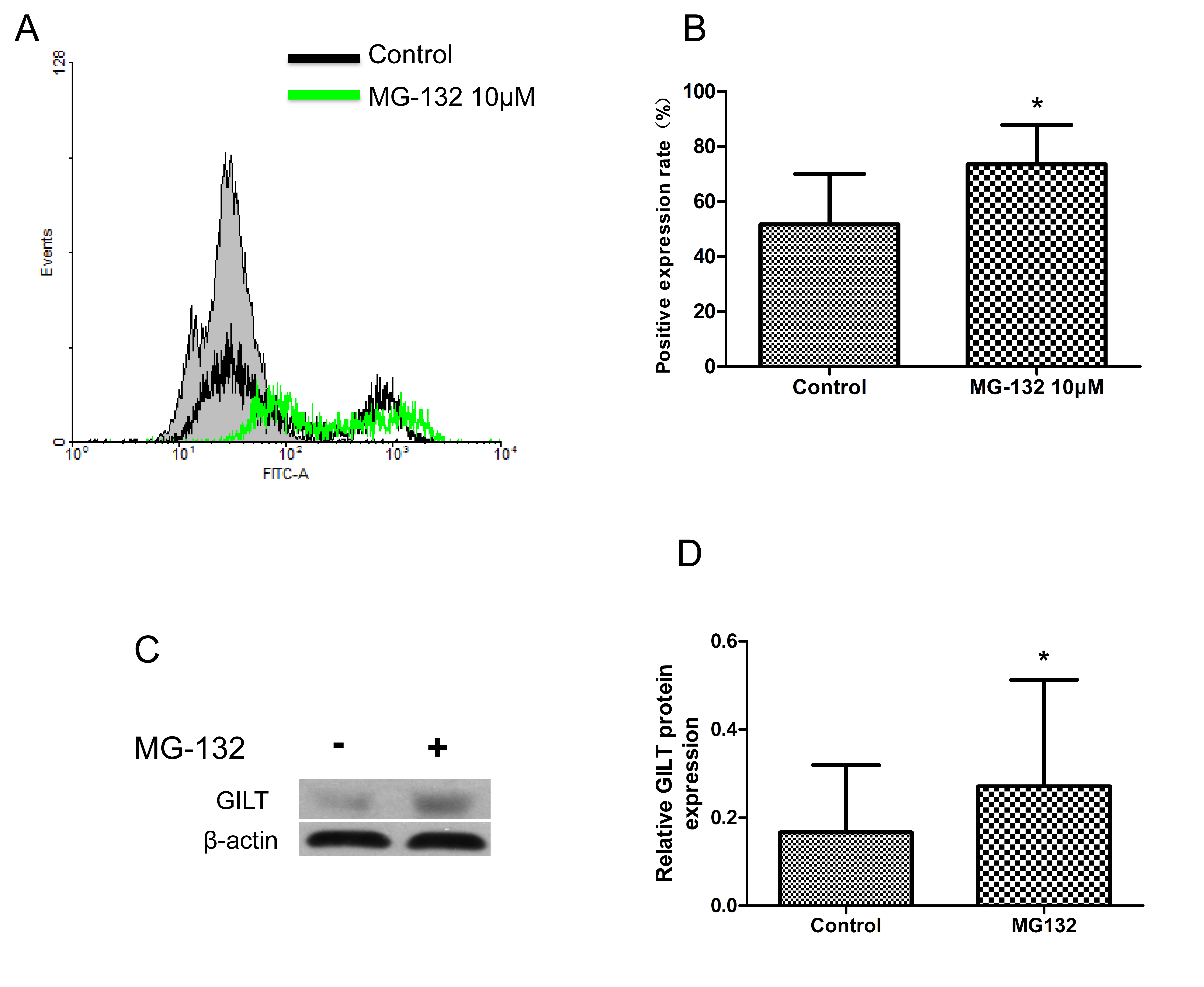

Supplement: Figure S1 — GILT protein changed in MCF-7 cells after treated with proteasomes inhibitor MG-132. (A) Flow cytometry (FCM) analysis of the changes of GILT protein level in breast cancer cells after threated with 10 µM MG-132 for 12 hours in MCF-7 cells. (B) The positive expression rate of GILT detected by FCS in cancer cells significantly increased from 51.7% to 73.6% after treated with MG-132 for 12 hours (P = 0.044). (C) Western blot analysis of the changes of GILT protein expression in breast cancer cells after threated with 10 µM MG-132 for 12 hours in MCF-7 cells. (D) Protein band density was analyzed with the Image J software. β-actin was used as the internal control. Data was normalized and expressed as means ± standard deviation (SD). And the relative protein expression level of GILT in cancer cells significantly increased after treated with MG-132 for 12 hours (P = 0.008). (TIF) [file pone.0109449.s001.tif]
